# LC-MS Analysis and Antifungal Activity of *Turnera subulata* Sm.

**DOI:** 10.3390/plants12020415

**Published:** 2023-01-16

**Authors:** Jacqueline Cosmo Andrade-Pinheiro, Celestina Elba Sobral de Souza, Daiany Alves Ribeiro, Andressa de Alencar Silva, Viviane Bezerra da Silva, Antonia Thassya Lucas dos Santos, Victor Juno Alencar Fonseca, Delmacia Gonçalves de Macêdo, Rafael Pereira da Cruz, José Weverton Almeida-Bezerra, Antonio Júdson Targino Machado, Thiago Sampaio de Freitas, Edy Sousa de Brito, Paulo Riceli Vasconcelos Ribeiro, José Galberto Martins da Costa, Henrique Douglas Melo Coutinho, Grażyna Kowalska, Rafał Rowiński, Radosław Kowalski, Maria Flaviana Bezerra Morais-Braga

**Affiliations:** 1 Pimenta Campus, Regional University of Cariri (URCA), Av. Cel Antônio Luis, 1161, Pimenta, Crato 63105-010, Brazil; 2 Laboratório de Bioensaios, Federal University of Cariri (UFCA), R. Olegário Emidio de Araujo, s/n, Centro, Brejo Santo 63260-000, Brazil; 3 Department of Botany, Federal University of Pernambuco (UFPE), Av. Prof. Moraes Rego, 1235, Recife 50670-901, Brazil; 4 Embrapa Agroindústria Tropical, Tropical R. Pernambuco, 2270-Pici, Fortaleza 60511-110, Brazil; 5 Department of Tourism and Recreation, University of Life Sciences in Lublin, 15 Akademicka Str., 20-950 Lublin, Poland; 6 Department of Analysis and Food Quality Assessment, University of Life Sciences in Lublin, 8 Skromna Str., 20-704 Lublin, Poland

**Keywords:** yeasts, antifungal, extract, virulence

## Abstract

Fungi of the *Candida* genus are responsible for invasive candidiasis, which affects people all over the world and has high mortality rates. This is due to their virulence factors, which give them great resistance and pathogenicity. In addition, the emergence of multidrug-resistant strains makes it difficult to treat these infections. In this way, natural products have emerged as an alternative to standard drugs, where plants known for their medicinal properties such as *Turnera subulata* become attractive to research. The present work aimed to analyze the ethanol extract of *Turnera subulata* leaves against standard strains of *Candida albicans*, *Candida krusei* and *Candida tropicalis* using broth microdilution techniques. The identification of the compounds in *T. subulata* leaves by LC-MS revealed the presence of a wide variety of substances such as carboxylic acids and terpenes, with flavonoids and fatty acids being more evident. The antifungal assays showed that the extract was not able to inhibit the growth of the tested strains at concentrations with a clinical relevance. However, at higher concentrations, it was able to inhibit the fungal dimorphism of *C. albicans* and *C. tropicalis*. It is possible that the *T. subulata* extract has potential as an inhibitor of fungal virulence factors without affecting the cell viability. Further research should be carried out in order to assess its inhibitory potential for other fungal virulence factors.

## 1. Introduction

Fungal infections caused by *Candida*, *Cryptococcus* and *Aspergillus* species kill thousands of people annually. This happens because these infections are difficult to treat and are often neglected. Although there are antifungal drugs that are widely used in medicine with relative effectiveness, mortality rates remain high as these microorganisms are capable of developing a resistance to this class of drugs [1,2].

The development of new drugs and diagnostic tools is important to avoid these problems and requires extensive knowledge about the biology of fungal pathogens, especially commensals, which, under favorable conditions, develop into difficult to treat infections [2]. Species such as *Candida albicans* and *Candida tropicalis* have characteristics that make them difficult to eradicate, such as the production of biofilms and fungal dimorphism; these are phenomena that are often associated with virulence, aiding in the colonization, tissue invasion and evasion of the immune system [3,4,5].

Researchers around the world have been looking for natural alternatives such as medicinal plants because, due their chemical constituents, they are seen as active therapeutic sources with the potential for the development of new drugs [6]. In addition, plant phytochemicals may be effective in combination therapies with commercial drugs, decreasing adverse effects and reversing fungal resistance to drugs [7,8].

The Passifloraceae family is represented by 12 genera and about 220 species and is distributed in the Americas and Africa [9,10,11]. In Brazil, the 2 largest genera of this family are found, *Piriqueta* and *Turnera*, which are distributed in 155 species [12,13]. The *Turnera* genus includes 135 species distributed in the tropical and subtropical regions of America and parts of Africa [10,11].

*Turnera subulata* Sm. (Passifloraceae) is a herbaceous plant frequently found in the North and Northeast of Brazil. Popularly known as “Chanana”, the species stands out for its medicinal properties, as reported in several studies evidencing its activities as an antibacterial, by modulating the action of drugs such as amikacin, neomycin and tobramycin against *Escherichia coli* [14] and gentamicin and kanamycin against *Staphylococcus aureus* [15]; as an antifungal, by modulating the action of metronidazole against *C. tropicalis* [16]; and as an anthelmintic against *Haemonchus contortus* in the early stages of development [17]. 

According to the literature, its composition contains flavonoids, alkaloids, tannins and phenolic compounds [18,19,20,21]; important classes that participate in the development of the biological activities of the species and that stand out in the investigation of bioactive compounds with a pharmaceutical potential. Therefore, and considering the medicinal importance of this species, in this work we aimed to evaluate the antifungal action of the ethanol extract of the leaves of *Turnera subulata* against the strains of *Candia* spp. as well as its inhibitory potential of fungal dimorphism.

## 2. Results

### 2.1. Identification of Chemical Composition

**Figure 1:**
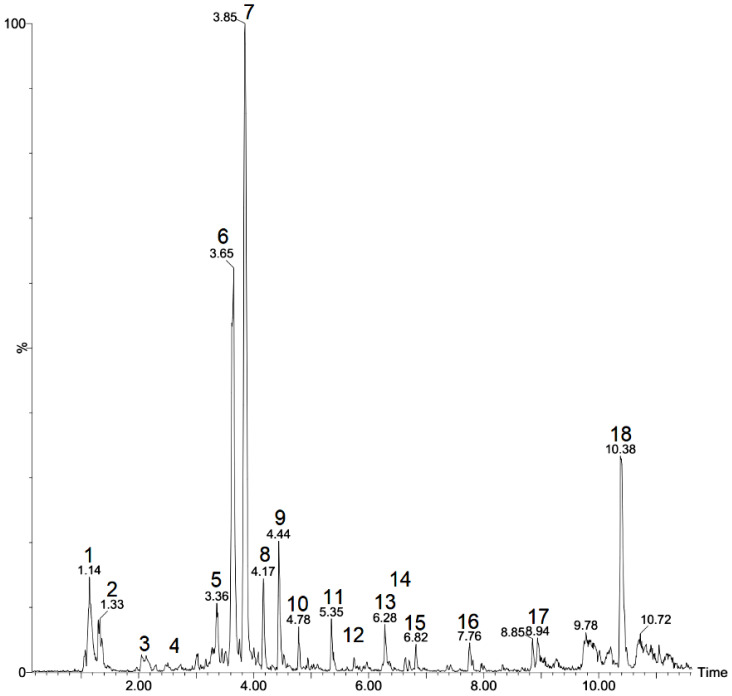
Chromatogram showing the negative mode of *T. subulata* leaves.

The identification of the compounds in *T. subulata* leaves revealed the presence of a wide variety of substances such as carboxylic acids and terpenes, with flavonoids and fatty acids being more evident, as represented in the chromatogram (Figure 1). Table 1 presents the chromatographic and mass spectral data such as the molecular ionic mass, retention time and fragmentation pattern for the compound identification.

Of the 18 peaks observed in the chromatograms, 12 were identified; among these, compounds 1, 2 and 3 exhibited [M-H]- at 191, 133 and 130 *m/z*, respectively. These were identified as quinic acid, malic acid and leucine by an authentic comparison of the pattern [22].

Compounds 6 and 7 showed a deprotonated [M-H]- ion at 593 *m/z* and 577 *m/z*, respectively. Compound 6 was identified as rhamnosyl isoorientin due to the presence of a fragment at 473 *m/z* corresponding with a loss of the C-hexose moiety. A fragment at 429 corresponded with a loss of the O-rhamnose moiety and a water molecule as a fragment ion at 327 *m/z* corresponded with the additional loss of the C-hexose moiety [23]. Compound 7, presenting fragments at 413 and 293 *m/z* that corresponded with C-glycosyl flavones and O glycosylated in the sugar portion, was identified as rhamnosyl vitexin [24].

Compound 9 presented a mass [M-H]- at 305 *m/z*; it was identified as a hexose derivative formed by the dehydration of disaccharides [25]. Compound 10, a deprotonated molecule with a mass [M-H]- at 187 *m/z*, showed a prominent fragmented ion in m/z due to the loss of the water portion and was identified as azelaic acid [26].

Compounds 11, 12 and 14 were identified as apigenin derivatives. Compound 11 produced a molecular anion in the apigenin fragment at 269 *m/z*, pointing to the presence of apigenin 7-O-neohesperidoside (rhoifolin). Compound 12 was identified as apigenin 7-O-rutinoside (isorhoifolin) due to the fragment ions at 431 (M-rhamnose) *m/z* and 269 (M-rutinose) *m/z* [27].

### 2.2. Antifungal Tests

Table 2 shows the results of the intrinsic activity performed by the broth microdilution technique. The inhibition potential of the products tested against *Candida* compared with the standard drug fluconazole was identified. The results showed that the products were able to inhibit 50% of the microorganism population (IC_50_) only at high concentrations and only for *C. albicans* and *C. krusei*; fluconazole alone was effective against the three strains tested, where *C. albicans* was inhibited by 16.70 µg/mL, *C. tropicalis* by 9.30 µg/mL and *C. krusei* by 133.32 µg/mL.

**Figure 2:**
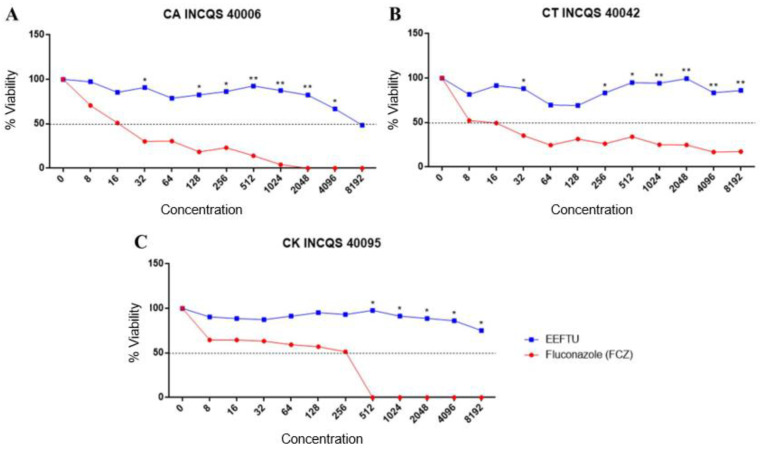
Antifungal effect of *Turnera subulata* ethanol extract (EELTS) compared with standard antifungal. FCZ: fluconazole. (**A**) CA: *Candida albicans* INCQS 40006; (**B**) CT: *Candida tropicalis* INCQS 40042; (**C**) CK: *Candida krusei* INCQS 40095. *p* < 0.05. * *p* < 0.1 and ** *p* < 0.01 when compared with the growth control.

The fungal viability curve (Figure 2) was constructed from the values obtained from an ELISA spectrophotometer. It was found that the ethanol extract of *Turnera subulata* (EELTS) against the *Candida* strains (CT INCQS 40006, CT INCQS 40042 and CK INCQS 40095) was above 8192 µg/mL, showing a slight reduction in the percentage of microorganisms on the cell viability curve. In the minimum fungicide concentration (MFC), the absence of EELTS results indicated a fungistatic effect on *Candida* (≥ 16,384 µg/mL).

**Figure 3:**
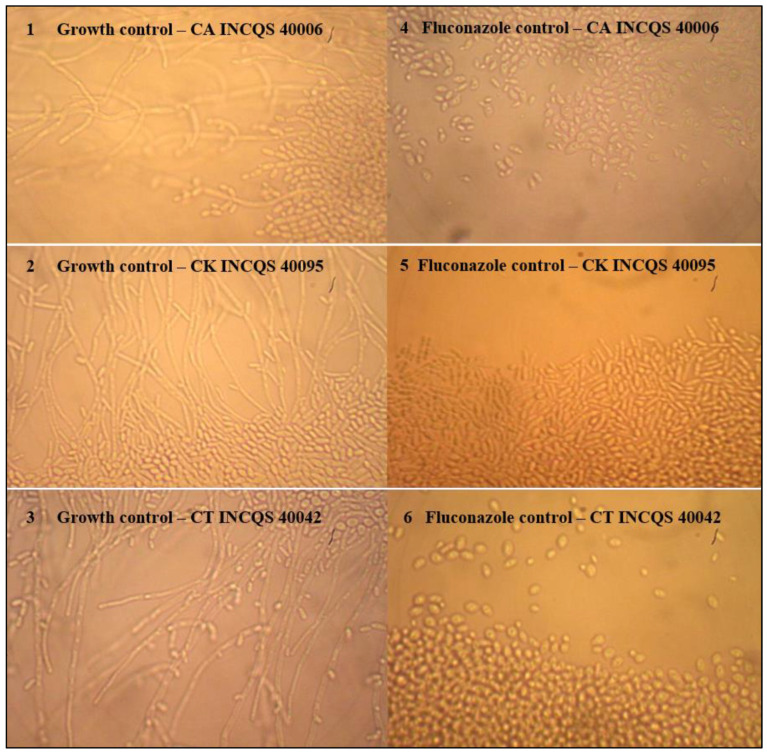
Growth control and fluconazole effect in *Candida* morphological transition assays. (**1**–**3**): *Candida* growth control; (**4**–**6**): effect of fluconazole. View from a 40 × objective. CA: *Candida albicans*; CK: *Candida krusei*; CT: *Candida tropicalis*.

The images presented in Figure 3 show the growth control and the control of the effect of fluconazole on the fungal dimorphism. In the micromorphology reading, it could be observed that the properly depleted medium stressed the *Candida* strains, driving the morphological transition and causing the emission of hyphae and pseudohyphae.

**Figure 4:**
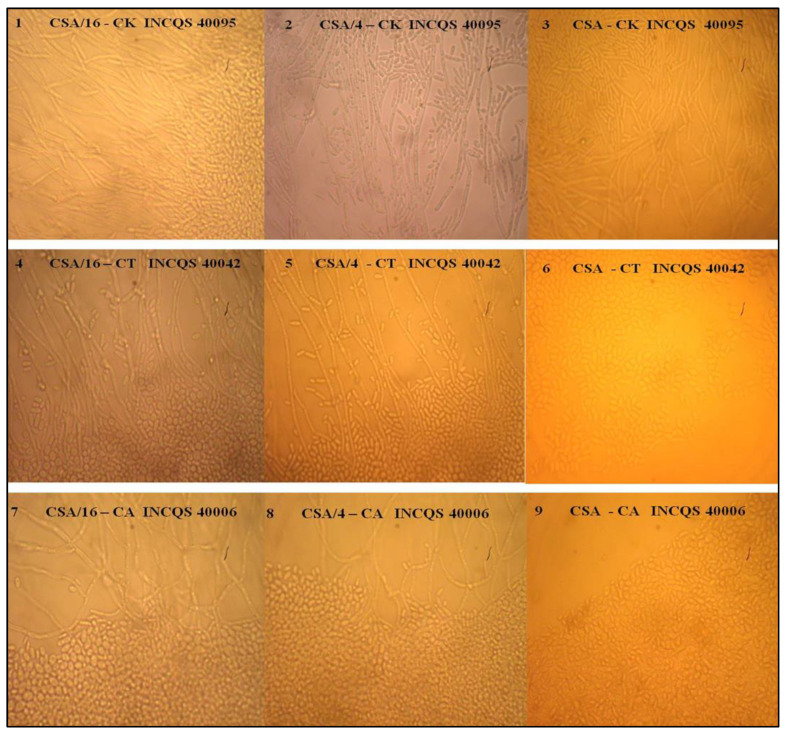
Effect of ethanol extract from *Turnera subulata* leaves on *Candida* morphological transition. CK: *Candida krusei*; CT: *Candida tropicalis*; CA: *Candida albicans*; INCQS: Instituto Nacional de Controle de Qualidade em Saúde. (**1**,**4**,**7**): concentrations of 512 µg/mL; (**2**,**5**,**8**): 2048 µg/mL; (**3**,**6**,**9**): 8192 µg/mL.

When cultivated in the growth medium added to the ethanolic extract of the leaves of *Turnera subulata*, *C. krusei* did not show any changes in its dimorphic potential at any of the concentrations tested. The presence of hyphae and pseudohyphae was recorded, as seen in Figure 4. For *C. albicans* and *C. tropicalis*, there was inhibition only at the highest HSA-8192 µg/mL concentration.

## 3. Discussion

Fungal infections affect people all over the world. In most of these infections, the isolated fungi are of the *Candida* genus, with *C. albicans* being the most common. However, many non-albicans *Candida* species are pathogenic. This pathogenicity is due to virulence factors such as the ability to develop biofilms, which gives them a great resistance. In addition, these fungi can develop a drug resistance [30,31]. Thus, natural products represent an alternative in the treatment of these infections, which can promote a reduction in the fungal virulence or even promote the action of drugs in combined therapies [32].

In a study carried out by Santos et al. [16] using an ethanol extract of *T. subulata* leaves, there was no clinically relevant antifungal activity against strains of *C. albicans* ATCC 40227, *C. krusei* ATCC 40147 and *C. tropicalis* ATCC 13803 when the MIC of the product was ≥1024 μg/mL. In addition, in a combined activity with drugs, the extract did not show any changes in the MIC when associated with amphotericin B and nystatin, but showed a potentiating effect of antifungal activity against *C. tropicalis* when associated with metronidazole.

In a study carried out by Morais [33], the crude extract as well as the hexane fractions and ethyl acetate from *T. subulata* also did not show a significant antifungal activity against strains of *C. albicans* ATCC 90028, *Candida dubliniensis* CBS 7987, *C. tropicalis* ATCC 13803, *Candida parapsilosis* ATCC 22019, *Candida glabrata* ATCC 2001, *C. krusei* ATCC 6258 and *Candida rugosa* ATCC 10571.

Similarly, the present work showed that EELTS did not show an antifungal activity against the three tested *Candida* strains, although flavonoids were the main component of the extract. It is known that flavonoids exhibit diverse biological activities, including antifungal activities [34]. These activities are extensively reported in the literature and, according to Jin et al. [35], flavonoids can act on the cell wall as well as biofilm formation and fungal dimorphism. This may elucidate the results obtained, where EELTS was able to inhibit the development of hyphae at the highest concentrations against *C. albicans* and *C. tropicalis*.

Fatty acids present in EELTS may also have contributed to the inhibition of fungal dimorphism in *C. albicans* and *C. tropicalis*. Studies show that fatty acids can reduce the virulence of these fungi such as biofilm formation, hyphae growth and cell aggregation [36]. The plasticity of the fungal cells of the genus *Candida* has been frequently associated with an increase in the virulence and, for this reason, there is great interest in researching the compounds capable of inhibiting these factors. It is more favorable to reduce the virulence of fungi without interfering with their cell viability, thus being able to prevent the development of resistance [3,5].

Interestingly, although the fluconazole activity was effective against *C. krusei*, it was still more resistant than *C. albicans* and *C. tropicalis*. According to Sampaio et al. [37], this difference in the antifungal activity of fluconazole between *Candida* species may be due to subtle changes between them, which give them resistance. In agreement, Arendrup and Patterson [38] reported that this resistance in *Candida* does not occur in the same way among their species. For *C. albicans*, the prolonged use of antifungals followed by recurrent infections such as chronic mucocutaneous candidiasis increases the chances of an acquired resistance. For several non-albicans *Candida* species such as *C. krusei*, there is less susceptibility to several classes of antifungals.

## 4. Materials and Methods

### 4.1. Plant Collection

Fresh leaves of *Turnera subulata* were collected from the Araripe National Forest (FLONA; Araripe Apodi) in a locality known as Barreiro Grande (07°21′ S and 039°28′ W), located in the municipality of Crato in the south of the State of Ceará (Brazil, Crato).

### 4.2. Preparation of Ethanol Extract

A total of 500 g of fresh leaves of *Turnera subulata* was crushed and then subjected to an exhaustive removal in 95% ethanol for 72 h. The extraction solution was subjected to solvent distillation on a rotary evaporator under a reduced pressure at an average temperature of 50 °C [39]. After distilling the solvent to dryness, the ethanolic extract of the fresh leaves of *Turnera umifolia* (EELTS) was obtained, with a percentage yield of 1.1%.

### 4.3. LC-MS Conditions

The analyses were performed using an Acquity UPLC (Waters, Milford, MA, USA) system coupled to a Xevo Quadrupole and Time-of-Flight mass system (QTOF, Water, Milford, MA, USA). A Waters Acquity BEH C18 column was used for the separation condition (150 mm × 2.1 mm; 1.7 μm) and set at 40 °C. An injection volume of a 5 μL aliquot of ethanolic extract was subjected to an exploratory gradient. The mobile phase was composed of deionized water (A) and acetonitrile (B) and both contained formic acid (0.1% *v*/*v*). The extracts were subjected to an exploratory gradient as follows: 2–95% B (15.0 min), 100% B (15.01–17.0 min) and 2% B (17.1–19.0 min), with a flow rate of 0.22 mL min^−1^.

Mass spectrometry analyses were performed using a mass spectrometer equipped with an ionization source (QTOF, Water, Milford, MA, USA) with an electrospray ionization source in the negative mode of ESI acquired in the range of 110–1200 Da. The optimized instrumental parameters were as follows (to negative): capillary voltage at 2.8 kV, cone voltage at 50 V, source temperature at 120 °C, desolvation temperature at 350 °C, flow cone gas at 20 L h^−1^ and desolvation gas flow at 500 L h^−1^. The mode of acquisition was MS^E^. The system was controlled using MassLynx 4.1 software (Waters Corporation, Milford, MA, USA).

### 4.4. Antifungal Assays

#### 4.4.1. Microorganisms

For the evaluation of the antifungal activity, three standard strains obtained from the Oswaldo Cruz Culture Collection of the National Institute for Quality Control in Health (INCQS) were used: *C. albicans* CA INCQS 40006; *C. krusei* CK INCQS 40095; and *C. tropicalis* CT INCQS 40042.

#### 4.4.2. Growth Media

A Sabouraud Dextrose Agar (SDA) medium purchased from HIMEDIA^®^ was prepared according to the manufacturer’s instructions. Sabouraud Dextrose Broth (CSD), purchased from KASVI^®^ and doubly concentrated, was used in the assays to evaluate the antifungal activity. For the analysis of fungal dimorphism, a potato dextrose agar (PDA) medium, purchased from Difco^®^, was used. The growth media were solubilized with distilled water and sterilized in an autoclave at 121 °C for 15 min.

#### 4.4.3. Inoculum Preparation

The strains were initially kept in test tubes containing SDA under refrigeration (8 °C). For the minimum inhibitory concentration (MIC) and minimum fungicide concentration (MFC) tests, the fungi were initially cultivated in Petri dishes containing SDA and incubated at 37 °C for 24 h (overnight). After this, the suspensions of microorganisms were prepared in tubes containing 4 mL of a sterile solution (0.9% NaCl). These suspensions were then shaken in a vortex mixer and the turbidity was compared and adjusted according to the 0.5 McFarland scale, which corresponded with an inoculum of approximately 10^5^ colony-forming units (mL–CFU/mL) [40].

#### 4.4.4. Drugs and Reagents

Dimethylsulfoxide (DMSO-Dynamic) was used to dilute the extract. The antifungal fluconazole at a dose of 150 mg (PRATI-Donaduzzi) was diluted in distilled water and used as a reference drug for the antifungal tests. In the preparation of the matrix solution of the extract, 0.15 g was weighed and then solubilized in 1 mL of DMSO. The extract and fluconazole were diluted again in sterile distilled water in order to obtain the desired concentration for the tests (16,384 µg/mL). The assay was performed using DMSO with a final concentration lower than 10% (the pilot assay performed in the lab indicated that DMSO concentrations lower than 10% did not affect the final results).

#### 4.4.5. Intrinsic Activity of the Antifungal Effect of EELTS and Fluconazole

An antifungal test with EELTS and fluconazole was performed using the broth microdilution technique and 96-well polystyrene plates. A total of 100 µL of a double-concentrated SDB medium was added to each well, plus the fungal suspension (10%). Subsequently, 100 µL of the natural product at a concentration of 16,384 µg/mL was deposited in the first well, from which the serial microdilution was carried out until the penultimate well; the concentrations ranged from 8192 to 8 µg/mL. The last well was reserved for the growth control. Controls for the sterility of the medium and the dilution of the natural product and fluconazole were also performed [41].

The plates were incubated at 37 °C for 24 h. After this period, they were taken to be read in an ELISA spectrophotometer device (Termoplate^®^) with a wavelength of 630 nm [42]. The results provided the minimum inhibitory concentration (MIC) of the tested products as well as the IC_50_. The tests were performed in quadruplicate.

#### 4.4.6. Determination of the Minimum Fungicide Concentration

With the aid of a sterile rod, the aliquots were transferred from each well of the MIC test plate, where the concentrations varied from 8192 to 8 µg/mL, to Petri dishes containing SDA. The plates were incubated at 37 °C for 24 h. After this period, the plates were checked for the growth of *Candida* colonies. The MFC was defined as the lowest concentration of the natural product capable of inhibiting the growth of fungal colonies [43].

#### 4.4.7. Effect of EELTS on the Fungal Dimorphism of *Candida* spp.

In the observation of the morphological alterations of the *Candida* strains against the EELTS extract, the technique of microculture for yeasts was used, using the depleted PDA medium in humid chambers. Intrinsic activity concentrations were considered, with concentrations of 8192 µg/mL being evaluated as HSA (a higher concentration assessed) as well as 2048 µg/mL (HSA/4) and 512 µg/mL (HSA/16). A total of 3 mL of the medium associated with the product tested was poured onto glass slides in a humid chamber. After the solidification of the medium, the yeast was seeded with the aid of a calibrated loop of 1 µL; two parallel streaks were then drawn, which were covered with sterile coverslips. The plates were incubated at 37 °C. After 24 h, the slides were observed under an optical microscope with a 40 × objective. A yeast growth control was performed as well as a fluconazole control for comparative purposes. The microcultures were photographed with an attached camera with a 5 x zoom. The tests were performed according to Sidrim and Rocha [44] and Mendes [45], with a few modifications.

### 4.5. Statistical Analysis

Quadruplicates were performed for each test and a two-way ANOVA analysis of variance with Fisher’s test was applied to each sample. The IC_50_ values were computed by a linear regression for the interpolation into standard curves relating to the percentage (%) of the growth values and the product concentration in μg/mL using GraphPad Prism software, version 5.0.

## 5. Conclusions

Although EELTS did not affect the cell viability of the three tested *Candida* strains, it was possible to observe, at high concentrations, an inhibitory effect on dimorphism in *C. albicans* and *C. tropicalis*. These results suggested that the product could act on these fungi directly on their virulence factors without affecting the cell viability. Further studies are needed to check its activity against other virulence factors such as biofilm formation. In addition, it is possible that this product could modulate the action of drugs, enhancing their activity.

## Figures and Tables

**Table 1:** Compounds identified in the leaves of *T. subulata* in the negative mode of ESI.

Peak N°	Rt Min	[M-H]- Observed	[M-H]- Calculated	Product Ions (MS/MS)	Empirical Formula	Ppm Error	Putative Identification	Ref.
1	1.14	191.0550	191.0556	-	C_7_H_11_O_6_	−3.1	Quinic acid *	[22]
2	1.33	133.0133	130.0137	-	C_4_H_5_O_5_	−3.0	Malic acid *	[22]
3	2.05	130.0863	130.0868	-	C_6_H_12_NO_2_	−3.8	Leucine *	[22]
4	3.03	409.1877	409.1862	179, 119	C_21_H_29_O_8_	3.7	Unknown	-
5	3.36	657.1106	657.1092	577, 341	C_30_H_25_O_17_	2.1	Unknown	-
6	3.65	593.1507	593.1506	473, 429, 369, 357, 327	C_27_H_29_O_15_	0.2	Rhamnosyl isoorientin	[23]
7	3.85	577.1560	577.1557	413, 293	C_27_H_29_O_14_	0.5	Rhamnosyl vitexin	[24]
8	4.17	553.3001	553.3013	507, 375	C_29_H_45_O_10_	−2.2	Unknown	-
9	4.44	305.1603	305.1600	179, 161	C_14_H_25_O_7_	1.0	Hexose derivative	[25]
10	4.78	187.0966	187.0970	125	C_9_H_15_O_4_	−2.1	Azelaic acid	[26]
11	5.35	577.1564	577.1557	269	C_27_H_29_O_14_	1.2	Apigenin 7-O-neohesperidoside	[27]
12	5.75	577.1558	577.1557	269, 431	C_27_H_29_O_14_	0.2	Apigenin 7-O-rutinoside	[27]
13	6.28	329.2322	329.2328	229, 211, 171	C_18_H_33_O_5_	−1.8	Trihydroxyoctadecaenoic acid	[28]
14	6.63	723.1807	723.1773	269	C_32_H_35_O_19_	4.7	Apigenin derivative	[27]
15	6.82	725.1939	725.1929	453, 271	C_32_H_37_O_19_	1.4	Unknown	
16	7.76	293.1766	293.1753	236, 221	C_17_H_25_O_4_	4.4	Unknown terpene	-
17	8.94	675.3601	675.3592	415, 397, 277, 235	C_33_H_55_O_14_	1.3	Digalactosylmono acylglycerol	[29]
18	10.38	555.2793	555.2805	255	C_28_H_43_O_11_	−2.2	Unknown fatty acid	-

* Comparison with authentic standard.

**Table 2:** Inhibitory concentrations of 50% (IC_50_) of microorganisms (μg/mL) by ethanol extract of the leaves of *Turnera subulata* and fluconazole.

Tested Products	Strains		
	CA INCQS 40006	CT INCQS 40042	CK INCQS 40095
Fluconazole (FCZ)	16.70 μg/mL	9.30 μg/mL	133.32 μg/mL
EELTS	7544.60 μg/mL	-	16,087.37 μg/mL

EELTS: ethanolic extract from the leaves of *Turnera subulata*; CA: *Candida albicans*; CT: *Candida tropicalis*; CK: *Candida krusei*; INCQS: Instituto Nacional de Controle de Qualidade em Saúde.

## Data Availability

Not applicable.
